# Supplementary material for: Machine learning identifies candidates for drug repurposing in Alzheimer’s disease
Source: Nat Commun. 2021 Feb 15;12:1033. doi: 10.1038/s41467-021-21330-0 (PMC7884393; doi:10.1038/s41467-021-21330-0)
Supplement: Supplementary file 1 — Supplementary Information [file 41467_2021_21330_MOESM1_ESM.pdf]

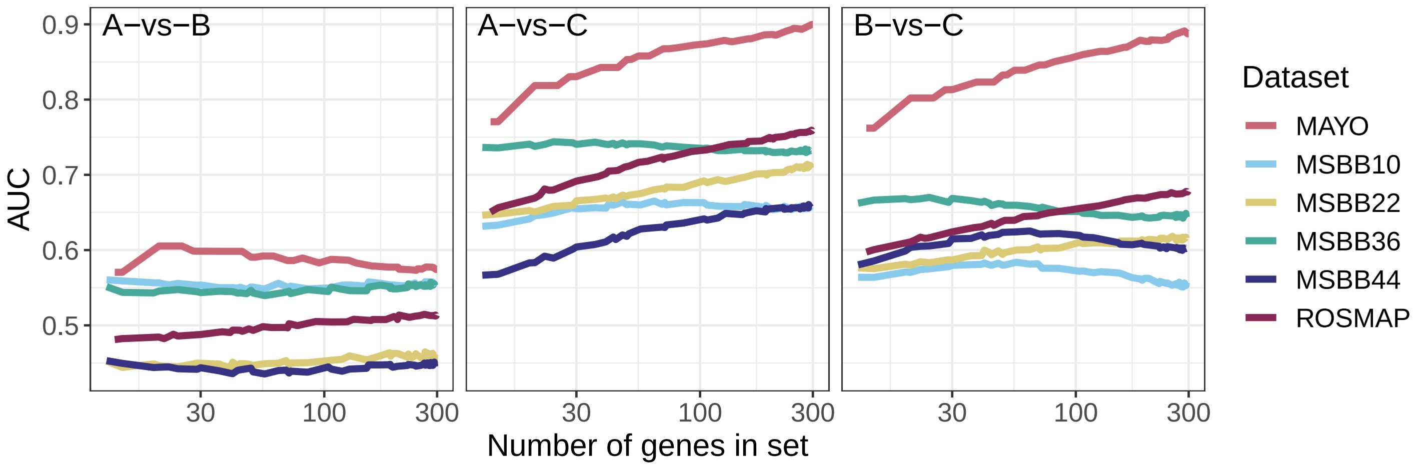

Supplementary Fig. 1: Performance of predictors trained on randomly-selected sets of genes plotted as a function of the set size. Performance was evaluated through leave-pair-out cross-validation and displayed as area under the ROC curve (AUC). The three panels correspond to binary classification tasks comparing early (A), intermediate (B) and late (C) disease stages. The color scheme, as introduced in Fig. 1b, denotes the dataset and brain region (specified as Brodmann Area) of samples used in each analysis.

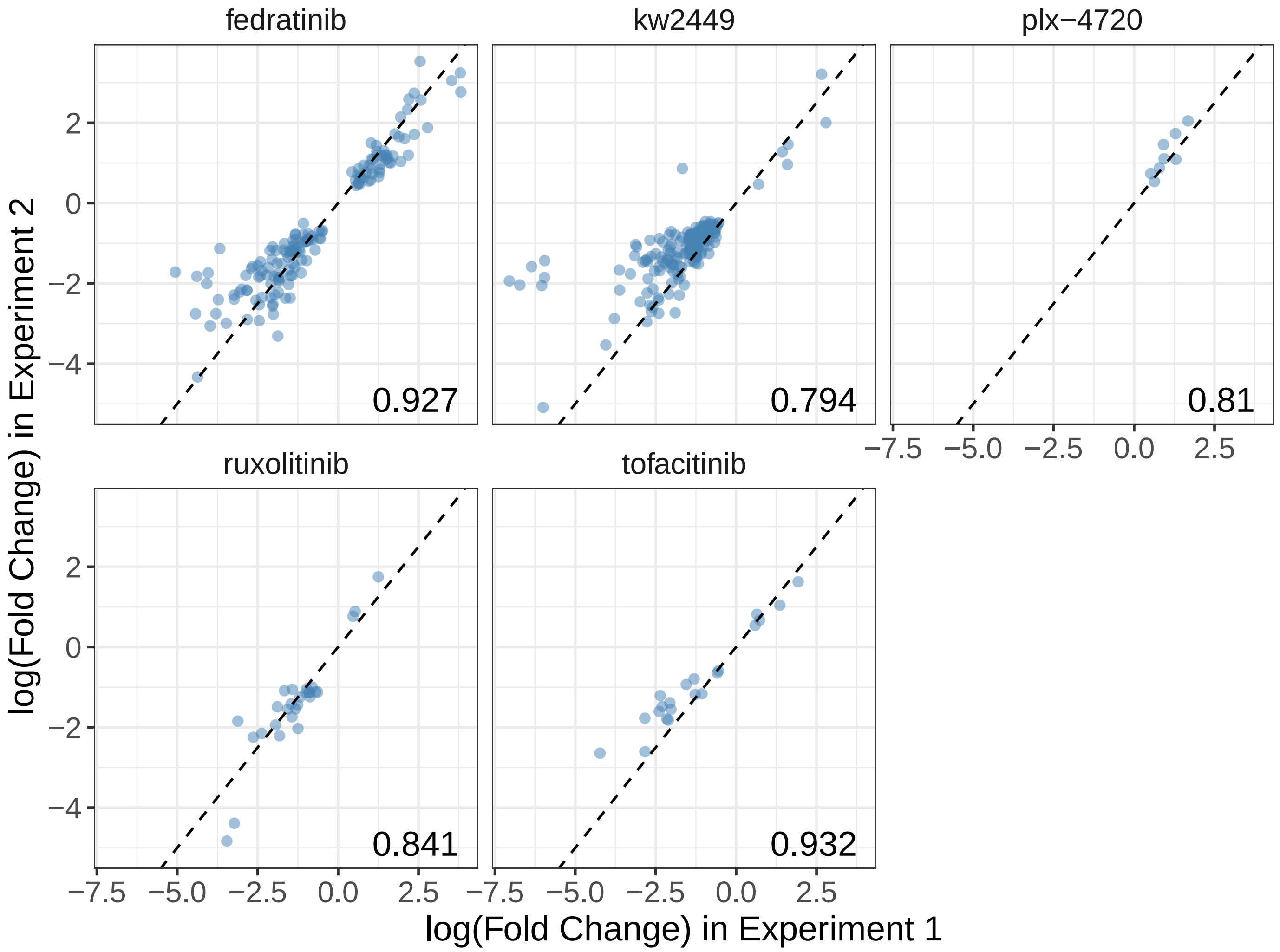

Supplementary Fig. 2: Concordance of treatment replicates across the two 3-DGE experiments. Shown are log-fold change values for all genes that were significantly (FDR ≤ 0.05) perturbed in both 3-DGE experiments. Spearman correlation between the two experiments is displayed in the bottom right corner of each panel.

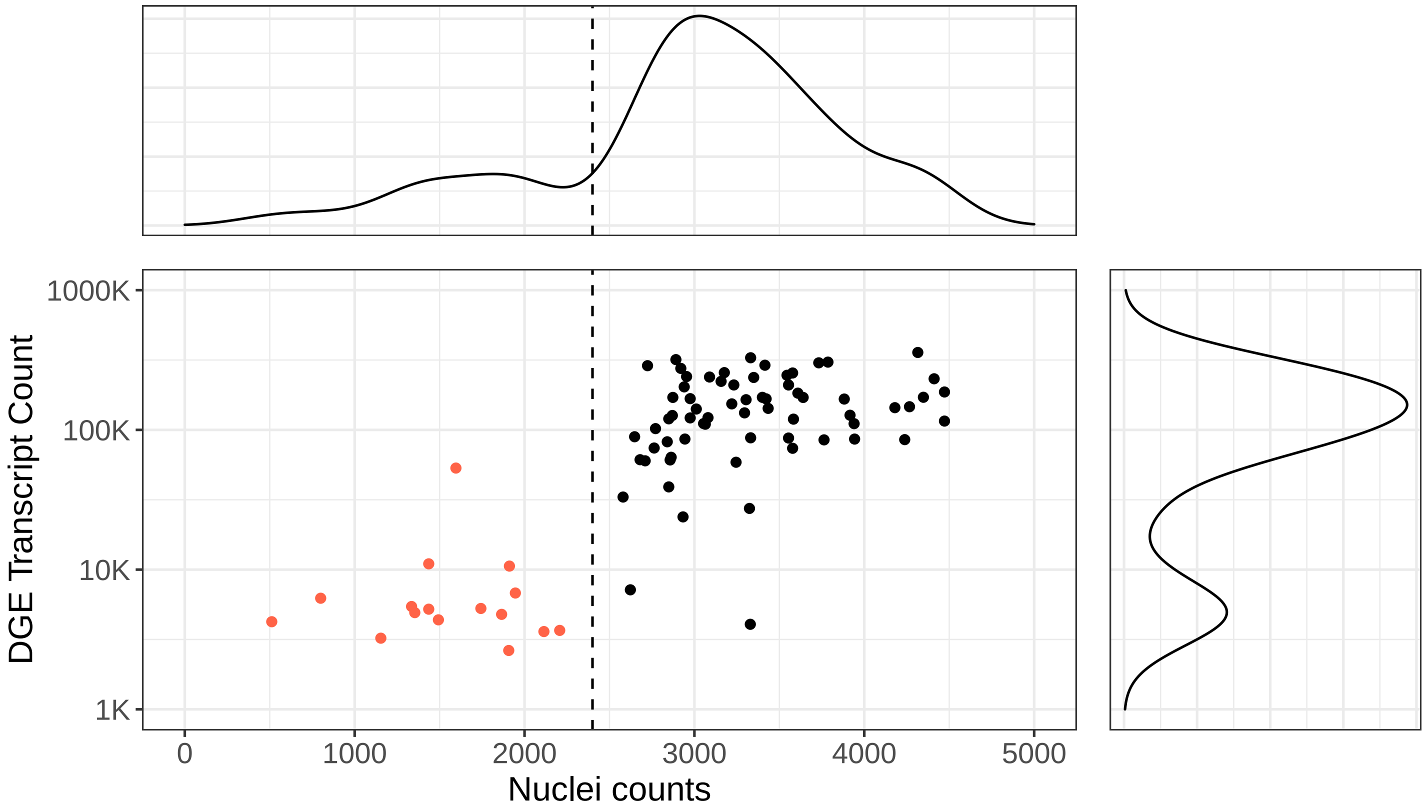

Supplementary Fig. 3: Assessment of compound toxicity. Nuclei counts estimated from microscopy images (x-axis) are plotted against mRNA abundance (y-axis). The mRNA abundance was computed as the total number of transcripts in the post-perturbational gene expression profile of the corresponding compound. Marginal distributions presented on the top and the right-hand side exhibit bi-modality, suggesting natural thresholds for determining compound neurotoxicity. A vertical dashed line is used to classify compounds into Toxic (red) and Non-Toxic (black) categories for Fig. 3.

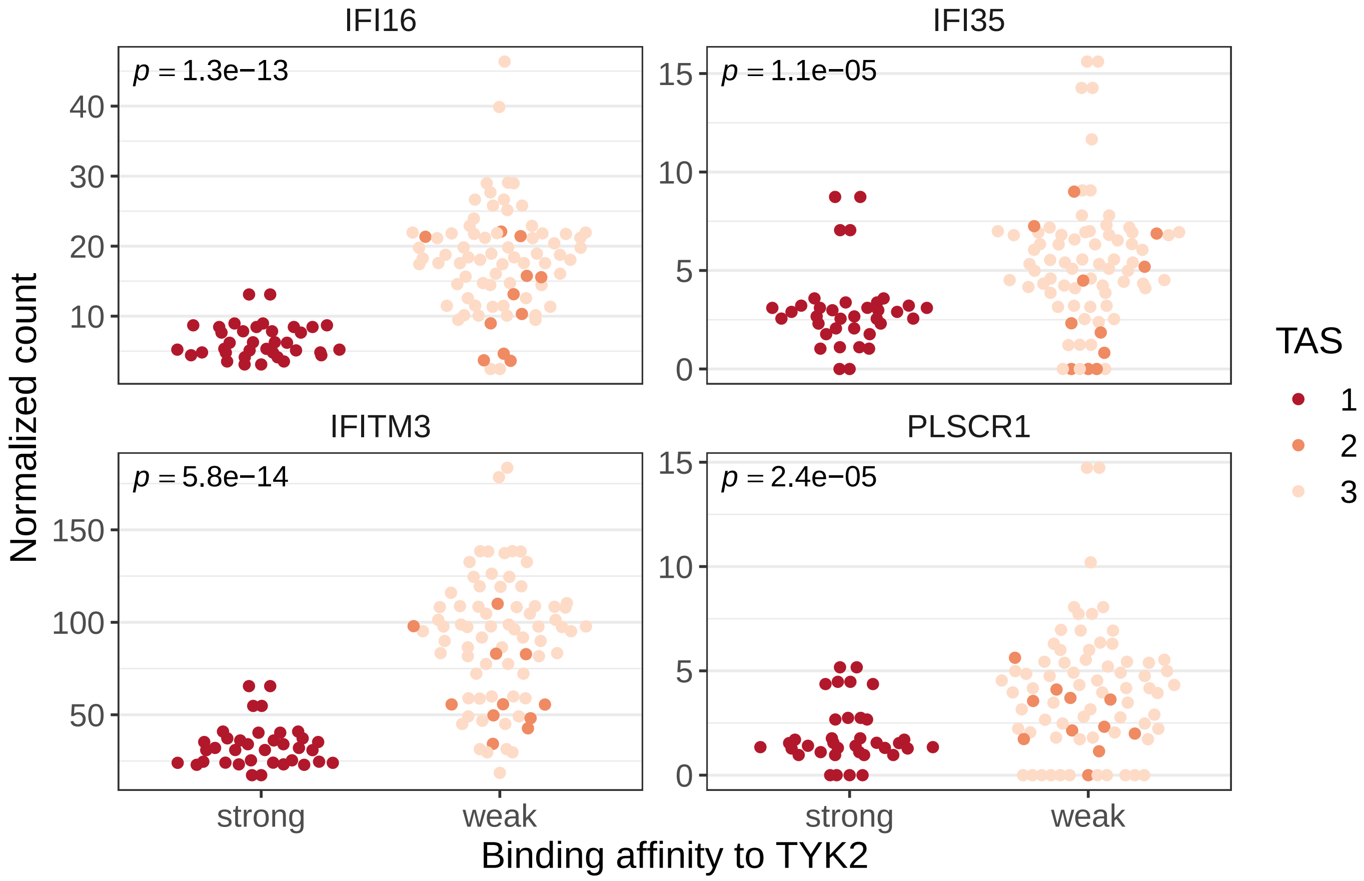

Supplementary Fig. 4: Raw expression values of selected interferon-stimulated genes. Each panel shows normalized transcript counts for a single interferon-stimulated gene (ISG). Individual points correspond to compounds that have a strong (TAS=1) or weak (TAS=2,3) binding to TYK2. Unadjusted p-values from two-sided Wilcoxon Rank Sum tests comparing the expression distributions between strong and weak binders are displayed in the top left corner of each panel.

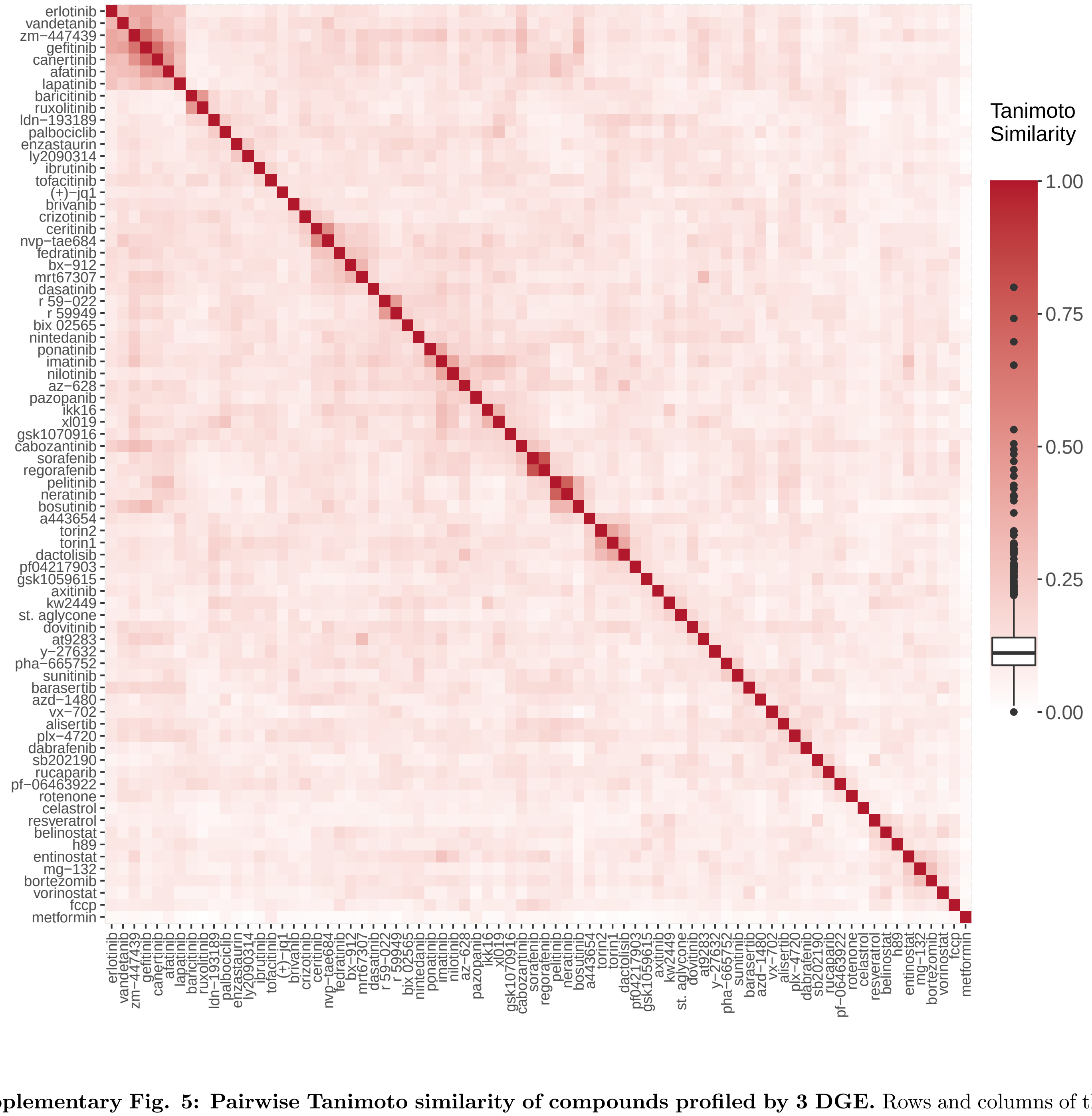

Supplementary Fig. 5: Pairwise Tanimoto similarity of compounds profiled by 3 DGE. Rows and columns of the heatmap are ordered according to hierarchical clustering. The boxplot in the legend shows the distribution of all non-diagonal values (i.e., non-self similarities).

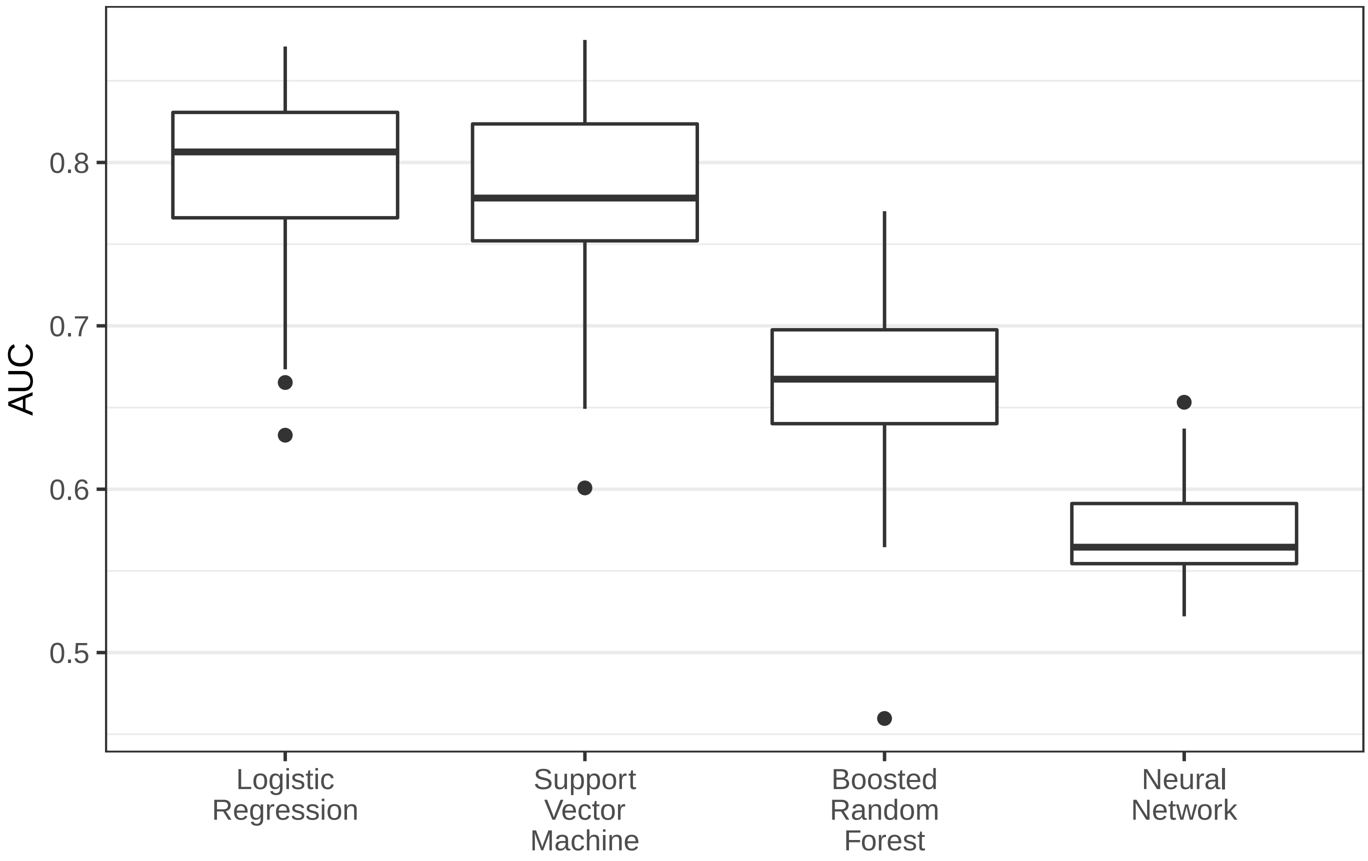

Supplementary Fig. 6: Comparison of machine learning methods implemented in DRIAD. Each method was used to train a model to distinguish early (A) vs. late (C) disease stages from AMP-AD gene expression data, where only genes associated with a given drug perturbation were considered by each predictor. Model performance was evaluated through leave-pair-out cross-validation and reported as Area under the ROC curve (AUC). The boxplots show how performance varies across 68 drug-associated gene lists that had at least 10 genes that were significantly (FDR $\leq$ 0.05) perturbed by the corresponding drug perturbations. The lower, middle and upper hinges of the boxplot correspond to the 25%, 50% (i.e., median) and 75% quantiles, respectively. The whiskers cover observations that are within 1.5 of the interquantile range (IQR) on each side. Ouliers beyond 1.5*IQR are shown as individual points.

Name	Sequence
E5V6NEXT	5’-iCiGiCACACTCTTTCCCTACACGACGCrGrGrG-3’
E3V6NEXT*<br>*BC6 sequences are below	/5Biosg/ACACTCTTTCCCTACACGACGCTCTTCCGATCT[BC6]NNNNNNNNNN<br>TTTTTTTTTTTTTTTTTTTTTTTTTTTTTTVN-3’
SINGV6	5’-/5Biosg/ACACTCTTTCCCTACACGACGC-3’
P5NEXTPT5	5’-AATGATACGGCGACCACCGAGATCTACACTCTTTCCCTACACGACGCTC<br>TTCCG*A*T*C*T-3’
P5_PCR	AATGATACGGCGACCACCGAG
P7_PCR	CAAGCAGAAGACGGCATACGAG
N701	5’ CAAGCAGAAGACGGCATACGAGATTCGCCTTAGTCTCGTGGGCTCGG
N703	5’ CAAGCAGAAGACGGCATACGAGATTTCTGCCTGTCTCGTGGGCTCGG

Name	Sequence	Name	Sequence	Name	Sequence	Name	Sequence
BC6-A01	AAAACT	BC6-E01	GTTTAT	BC6-I01	GCTAGA	BC6-M01	CGGTGG
BC6-A02	AAAATC	BC6-E02	GTTTTA	BC6-I02	GCTTAC	BC6-M02	CGTCCG
BC6-A03	AAAGTT	BC6-E03	TAAAGT	BC6-I03	GGGATT	BC6-M03	CGTGGG
BC6-A04	AAATAC	BC6-E04	TAAATG	BC6-I04	GTACAC	BC6-M04	CTCCCG
BC6-A05	AAATTG	BC6-E05	TAAGAT	BC6-I05	GTTCGA	BC6-M05	CTGCGG
BC6-A06	AACAAT	BC6-E06	TAAGTA	BC6-I06	TAGTGG	BC6-M06	CTGGCG
BC6-A07	AAGATT	BC6-E07	TAATGA	BC6-I07	TCTGCA	BC6-M07	GACCGC
BC6-A08	AAGTAT	BC6-E08	TAATTC	BC6-I08	TTCCTC	BC6-M08	GACGCC
BC6-A09	AATACA	BC6-E09	TACTAT	BC6-I09	CCAACC	BC6-M09	GAGCGG
BC6-A10	AATAGT	BC6-E10	TACTTA	BC6-I10	CCTTCC	BC6-M10	GAGGCG
BC6-A11	AATCTT	BC6-E11	TAGTAA	BC6-I11	GTACCG	BC6-M11	GCACGG
BC6-A12	AATGAT	BC6-E12	TAGTTT	BC6-I12	ACCCCC	BC6-M12	GCAGCG
BC6-A13	AATTCT	BC6-E13	TATAGA	BC6-I13	ACCGGC	BC6-M13	GCCAGG
BC6-A14	AATTGA	BC6-E14	TATATC	BC6-I14	ACGCCG	BC6-M14	GCCCAG
BC6-A15	ACAATA	BC6-E15	TATGAA	BC6-I15	ACGGGG	BC6-M15	GCCCTC
BC6-A16	ACATAA	BC6-E16	TATGTT	BC6-I16	AGCCCG	BC6-M16	GCCGAC
BC6-A17	ACTTAT	BC6-E17	TATTGT	BC6-I17	AGCGGG	BC6-M17	GCCGTG
BC6-A18	ACTTTA	BC6-E18	TATTTG	BC6-I18	AGGCCC	BC6-M18	GCCTCG
BC6-A19	AGATTA	BC6-E19	TCATAT	BC6-I19	AGGGGC	BC6-M19	GCGAGC
BC6-A20	AGTAAT	BC6-E20	TCATTA	BC6-I20	CACCCC	BC6-M20	GCGCAC
BC6-A21	ATAAAC	BC6-E21	TCTTAA	BC6-I21	CACGGC	BC6-M21	GCGCTG
BC6-A22	ATAACA	BC6-E22	TGAAAT	BC6-I22	CAGCCG	BC6-M22	GCGGAG
BC6-A23	ATACAA	BC6-E23	TGATTT	BC6-I23	CAGGGG	BC6-M23	GCGGTC
BC6-A24	ATACTT	BC6-E24	TGTAAA	BC6-I24	CCACCG	BC6-M24	GCGTCC
BC6-B01	AAACAT	BC6-F01	TAAAAC	BC6-J01	GGACAT	BC6-N01	CGTCGC
BC6-B02	AAACTA	BC6-F02	TAAACA	BC6-J02	GGCAAT	BC6-N02	CGTGCC
BC6-B03	AAATCA	BC6-F03	TAACAA	BC6-J03	GTCAAG	BC6-N03	CTCCGC
BC6-B04	AAATGT	BC6-F04	TAACTT	BC6-J04	GTGACT	BC6-N04	CTCGGG
BC6-B05	AACATA	BC6-F05	TAATAG	BC6-J05	TCCAAC	BC6-N05	CTGGGC
BC6-B06	AACTAA	BC6-F06	TAATCT	BC6-J06	TCGAAG	BC6-N06	GACCCG
BC6-B07	AAGTTA	BC6-F07	TACAAA	BC6-J07	TTGTCC	BC6-N07	GACGGG
BC6-B08	AATAAC	BC6-F08	TACATT	BC6-J08	TTTGGC	BC6-N08	GAGCCC
BC6-B09	AATATG	BC6-F09	TAGAAT	BC6-J09	CTCTCC	BC6-N09	GAGGGC
BC6-B10	AATCAA	BC6-F10	TAGATA	BC6-J10	GGACCA	BC6-N10	GCACCC
BC6-B11	AATGTA	BC6-F11	TATAAG	BC6-J11	ACCCGG	BC6-N11	GCAGGC
BC6-B12	AATTAG	BC6-F12	TATACT	BC6-J12	ACCGCG	BC6-N12	GCCACC
BC6-B13	AATTTC	BC6-F13	TATCAT	BC6-J13	ACGCGC	BC6-N13	GCCCCT
BC6-B14	ACAAAT	BC6-F14	TATCTA	BC6-J14	ACGGCC	BC6-N14	GCCCGA
BC6-B15	ACTAAA	BC6-F15	TATTAC	BC6-J15	AGCCGC	BC6-N15	GCCGCA
BC6-B16	ACTATT	BC6-F16	TATTCA	BC6-J16	AGCGCC	BC6-N16	GCCGGT
BC6-B17	AGAATT	BC6-F17	TCAAAA	BC6-J17	AGGCGG	BC6-N17	GCCTGC
BC6-B18	AGATAT	BC6-F18	TCAATT	BC6-J18	AGGGCG	BC6-N18	GCGACG
BC6-B19	AGTATA	BC6-F19	TCTAAT	BC6-J19	CACCGG	BC6-N19	GCGCCA
BC6-B20	AGTTAA	BC6-F20	TCTATA	BC6-J20	CACGCG	BC6-N20	GCGCGT
BC6-B21	ATAAGT	BC6-F21	TGAATA	BC6-J21	CAGCGC	BC6-N21	GCGGCT
BC6-B22	ATAATG	BC6-F22	TGATAA	BC6-J22	CAGGCC	BC6-N22	GCGGGA

BC6-B23	ATAGAT	BC6-F23	TGTATT	BC6-J23	CCACGC	BC6-N23	GCGTGG
BC6-B24	ATAGTA	BC6-F24	TGTTAT	BC6-J24	CCAGGG	BC6-N24	GCTCCG
BC6-C01	ATATAG	BC6-G01	TGTTTA	BC6-K01	CCCACG	BC6-O01	GCTCGC
BC6-C02	ATATCT	BC6-G02	TTAAAG	BC6-K02	CCCAGC	BC6-O02	GCTGCC
BC6-C03	ATCAAA	BC6-G03	TTAATC	BC6-K03	CCCCGT	BC6-O03	GGAGCC
BC6-C04	ATCATT	BC6-G04	TTACAT	BC6-K04	CCCCTG	BC6-O04	GGAGGG
BC6-C05	ATGAAT	BC6-G05	TTAGTT	BC6-K05	CCCTGG	BC6-O05	GGCCAC
BC6-C06	ATGATA	BC6-G06	TTATAC	BC6-K06	CCGAGG	BC6-O06	GGCGAG
BC6-C07	ATTACT	BC6-G07	TTATTG	BC6-K07	CCGGAC	BC6-O07	GGCGTC
BC6-C08	ATTAGA	BC6-G08	TTCAAT	BC6-K08	CCGGCA	BC6-O08	GGCTCC
BC6-C09	ATTCTA	BC6-G09	TTGAAA	BC6-K09	CCGTCG	BC6-O09	GGGCAG
BC6-C10	ATTGAA	BC6-G10	TTGATT	BC6-K10	CCGTGC	BC6-O10	GGGCCT
BC6-C11	ATTTCA	BC6-G11	TTTACA	BC6-K11	CCTGGC	BC6-O11	GGGGAC
BC6-C12	ATTTGT	BC6-G12	TTTAGT	BC6-K12	CGACCC	BC6-O12	GGGGCA
BC6-C13	CAAATA	BC6-G13	TTTCTT	BC6-K13	CGAGGC	BC6-O13	GGGTCG
BC6-C14	CAATAA	BC6-G14	TTTGTA	BC6-K14	CGCACC	BC6-O14	GGGTGC
BC6-C15	CATTAT	BC6-G15	TTTTGA	BC6-K15	CGCCCT	BC6-O15	GGTGGC
BC6-C16	CATTTA	BC6-G16	TCTTTC	BC6-K16	CGCCGA	BC6-O16	GTCCCC
BC6-C17	CTATAT	BC6-G17	AGACCT	BC6-K17	CGCGCA	BC6-O17	GTGCGC
BC6-C18	CTATTA	BC6-G18	AGGGAT	BC6-K18	CGCGGT	BC6-O18	GTGGCC
BC6-C19	CTTTAA	BC6-G19	CACCAA	BC6-K19	CGCTGC	BC6-O19	TCCCGC
BC6-C20	GAAATT	BC6-G20	CAGTCA	BC6-K20	CGGACG	BC6-O20	TCCGGG
BC6-C21	GATAAT	BC6-G21	CCACAT	BC6-K21	CGGCCA	BC6-O21	TCGGGC
BC6-C22	GATATA	BC6-G22	CCGATT	BC6-K22	CGGCGT	BC6-O22	TGCCCC
BC6-C23	GTAATA	BC6-G23	CTAGTG	BC6-K23	CGGGCT	BC6-O23	TGGCCG
BC6-C24	GTATAA	BC6-G24	CTTCTG	BC6-K24	CGGGGA	BC6-O24	TGGCGC
BC6-D01	ATATGA	BC6-H01	TTAACT	BC6-L01	CCCCAC	BC6-P01	GCTGGG
BC6-D02	ATATTC	BC6-H02	TTAAGA	BC6-L02	CCCCCA	BC6-P02	GGACGC
BC6-D03	ATCTAT	BC6-H03	TTACTA	BC6-L03	CCCGAG	BC6-P03	GGCACG
BC6-D04	ATCTTA	BC6-H04	TTAGAA	BC6-L04	CCCGGA	BC6-P04	GGCAGC
BC6-D05	ATGTAA	BC6-H05	TTATCA	BC6-L05	CCGCAG	BC6-P05	GGCGCT
BC6-D06	ATTAAG	BC6-H06	TTATGT	BC6-L06	CCGCGA	BC6-P06	GGCGGA
BC6-D07	ATTATC	BC6-H07	TTCATA	BC6-L07	CCGGGT	BC6-P07	GGGACC
BC6-D08	ATTCAT	BC6-H08	TTCTAA	BC6-L08	CCGGTG	BC6-P08	GGGAGG
BC6-D09	ATTGTT	BC6-H09	TTGTTA	BC6-L09	CCTCGG	BC6-P09	GGGCGA
BC6-D10	ATTTAC	BC6-H10	TTTAAC	BC6-L10	CCTGCG	BC6-P10	GGGCTC
BC6-D11	ATTTTG	BC6-H11	TTTATG	BC6-L11	CGACGG	BC6-p11	ATGTTT
BC6-D12	CAAAAT	BC6-H12	TTTCAA	BC6-L12	CGAGCG	BC6-P12	GGGGTG
BC6-D13	CATAAA	BC6-H13	TTTTAG	BC6-L13	CGCAGG	BC6-P13	GGTCCC
BC6-D14	CATATT	BC6-H14	TTTTCT	BC6-L14	CGCCAG	BC6-P14	GGTGCG
BC6-D15	CTAAAA	BC6-H15	TTGGAT	BC6-L15	CGCCTC	BC6-P15	GTCGCG
BC6-D16	CTAATT	BC6-H16	ACCGTA	BC6-L16	CGCGAC	BC6-P16	GTCGGC
BC6-D17	CTTAAT	BC6-H17	ATCGAG	BC6-L17	CGCGTG	BC6-P17	GTGGGG
BC6-D18	CTTATA	BC6-H18	CAAGCT	BC6-L18	CGCTCG	BC6-P18	TCCCCG
BC6-D19	GAATAT	BC6-H19	CATCAG	BC6-L19	CGGAGC	BC6-P19	TCGCGG
BC6-D20	GAATTA	BC6-H20	CATGGT	BC6-L20	CGGCAC	BC6-P20	TCGGCG
BC6-D21	GATTAA	BC6-H21	CGACTT	BC6-L21	CGGCTG	BC6-P21	TGCGCG
BC6-D22	GTAAAT	BC6-H22	CGATTG	BC6-L22	CGGGAG	BC6-P22	TGCGGC
BC6-D23	GTTAAA	BC6-H23	GAAGAC	BC6-L23	CGGGTC	BC6-P23	TGGGCC
BC6-D24	GTTATT	BC6-H24	GATCGT	BC6-L24	CGGTCC	BC6-P24	TGGGGG

Supplementary Table 1: The complete list of primers used for 3' Digital Gene Expression.
