# Supplementary material for: Machine learning identifies candidates for drug repurposing in Alzheimer’s disease
Source: Nat Commun. 2021 Feb 15;12:1033. doi: 10.1038/s41467-021-21330-0 (PMC7884393; doi:10.1038/s41467-021-21330-0)
Supplement: Supplementary file 5 — Reporting Summary [file 41467_2021_21330_MOESM5_ESM.pdf]

nature research

Corresponding author(s): albers.mark@mgh.harvard.edu, artem_sokolov@hms.harvard.edu

Last updated by author(s): Jan 11, 2021

Reporting Summary

Nature Research wishes to improve the reproducibility of the work that we publish. This form provides structure for consistency and transparency in reporting. For further information on Nature Research policies, see our Editorial Policies and the Editorial Policy Checklist.

Statistics

For all statistical analyses, confirm that the following items are present in the figure legend, table legend, main text, or Methods section.

n/a	Confirmed	
☐	☒	The exact sample size (n) for each experimental group/condition, given as a discrete number and unit of measurement
☐	☒	A statement on whether measurements were taken from distinct samples or whether the same sample was measured repeatedly
☐	☒	The statistical test(s) used AND whether they are one- or two-sided<br>*Only common tests should be described solely by name; describe more complex techniques in the Methods section.*
☐	☒	A description of all covariates tested
☐	☒	A description of any assumptions or corrections, such as tests of normality and adjustment for multiple comparisons
☐	☒	A full description of the statistical parameters including central tendency (e.g. means) or other basic estimates (e.g. regression coefficient) AND variation (e.g. standard deviation) or associated estimates of uncertainty (e.g. confidence intervals)
☐	☒	For null hypothesis testing, the test statistic (e.g. F, t, r) with confidence intervals, effect sizes, degrees of freedom and P value noted<br>*Give P values as exact values whenever suitable.*
☒	☐	For Bayesian analysis, information on the choice of priors and Markov chain Monte Carlo settings
☒	☐	For hierarchical and complex designs, identification of the appropriate level for tests and full reporting of outcomes
☐	☒	Estimates of effect sizes (e.g. Cohen's d, Pearson's r), indicating how they were calculated

Our web collection on statistics for biologists contains articles on many of the points above.

Software and code

Policy information about availability of computer code

Data collection	Device: HP D300e<br>Software v: 3.3.2
Data analysis	R 3.6.2, tidyverse 1.3.0, edgeR 3.26.5, https://github.com/labsyspharm/DRIAD, https://github.com/labsyspharm/DRIADrc

For manuscripts utilizing custom algorithms or software that are central to the research but not yet described in published literature, software must be made available to editors and reviewers. We strongly encourage code deposition in a community repository (e.g. GitHub). See the Nature Research guidelines for submitting code & software for further information.

Data

Policy information about availability of data

All manuscripts must include a data availability statement. This statement should provide the following information, where applicable:
- Accession codes, unique identifiers, or web links for publicly available datasets
- A list of figures that have associated raw data
- A description of any restrictions on data availability

Raw post-perturbational gene expression data for the 80 compounds profiled in this study, the associated gene lists, drug toxicity data and all relevant metadata have been uploaded to Synapse (doi: 10.7303/syn18488020). Raw sequences were also deposited to the NCBI Sequence Read Archive (SRA) under the accession number SRP301436. Additional datasets analyzed during the current study are available in the Synapse repository, https://adknowledgeportal.synapse.org/.

Field-specific reporting

Please select the one below that is the best fit for your research. If you are not sure, read the appropriate sections before making your selection.

- [x] Life sciences
- [ ] Behavioural & social sciences
- [ ] Ecological, evolutionary & environmental sciences

For a reference copy of the document with all sections, see nature.com/documents/nr-reporting-summary-flat.pdf

Life sciences study design

All studies must disclose on these points even when the disclosure is negative.

Sample size	Every drug-associated gene list evaluated in the study was compared against 1,000 randomly-selected gene lists of equal length. The choice of 1,000 was motivated by the desire to have empirical p values with three significant digits to allow for effective ranking of drugs.
Data exclusions	We chose to exclude MAYO data from most analyses performed in the study, due to an unknown batch effect (Suppl. Fig 1)
Replication	The 80 drugs were profiled across two 384-well plates. Five of the of the drugs were included in both plates to establish reproducibility. We observed strong concordance in measured gene expression for these five reference points (Suppl. Fig 2), signaling that our attempts at replication were successful.
Randomization	All machine learning predictors were evaluated in cross-validation on pairs of brain specimens (one specimen from each disease category). The pairs were matched by age, to alleviate any confounding effects due to age.
Blinding	Blinding was not possible because drug signatures were matched directly to neuropathological stage of postmortem brain specimens.

Reporting for specific materials, systems and methods

We require information from authors about some types of materials, experimental systems and methods used in many studies. Here, indicate whether each material, system or method listed is relevant to your study. If you are not sure if a list item applies to your research, read the appropriate section before selecting a response.

Materials & experimental systems

n/a	Involved in the study	
[x]	[ ]	Antibodies
[ ]	[x]	Eukaryotic cell lines
[x]	[ ]	Palaeontology and archaeology
[x]	[ ]	Animals and other organisms
[x]	[ ]	Human research participants
[x]	[ ]	Clinical data
[x]	[ ]	Dual use research of concern

Methods

n/a	Involved in the study	
[x]	[ ]	ChIP-seq
[x]	[ ]	Flow cytometry
[x]	[ ]	MRI-based neuroimaging

Eukaryotic cell lines

Policy information about cell lines

Cell line source(s)	ReNcell VM, Millipore, Billerica, MA (catalog # SCC008)
Authentication	Cell lines were authenticated by immunofluorescence staining for markers of glial cell types and by mass spectrometry proteomics profiling (over 9000 proteins) across maturation to establish that they were indeed human neural progenitors (published in PMID: 30778261). No STR profiling was performed.
Mycoplasma contamination	Cells tested negative for Mycoplasma.
Commonly misidentified lines (See ICLAC register)	We did not use any commonly misidentified cell lines.
