# Supplementary material for: Machine learning identifies candidates for drug repurposing in Alzheimer’s disease
Source: Nat Commun. 2021 Feb 15;12:1033. doi: 10.1038/s41467-021-21330-0 (PMC7884393; doi:10.1038/s41467-021-21330-0)
Supplement: Supplementary file 7 — Description of Additional Supplementary Files [file 41467_2021_21330_MOESM7_ESM.pdf]

Title: Supplementary Data 1:

Description: A ranking of drug targets by their contribution to the significant polypharmacology effects among the top-performing compounds. The columns correspond to targets, the number of pairs they appear in, whether those pairs are primarily positive or negative interactions, and the overall p-value computed by aggregating p-values from individual two-sided Wilcoxon Rank Sum tests using the Brown's method. Additional adjustment for multiple hypothesis testing was performed using the Benjamini Hochberg method.

Title: Supplementary Data 2:

Description: Gene sets from the literature used to evaluate the DRIAD framework in Fig. 1c.

Title: Supplementary Data 3:

Description: Eighty compounds profiled in differentiated neuroprogenitor cell cultures. Each compound is annotated with its LINCS identifier, nominal target, approval status, toxicity, and strength of association with disease severity in ROSMAP and MSBB datasets (presented as HMP, harmonic mean p-value). Five of the compounds were profiled in two separate 3' Digital Gene Expression (DGE) experiments.
